# Clinical Features of Optic Disc Drusen in an Ophthalmic Genetics Cohort

**DOI:** 10.1155/2020/5082706

**Published:** 2020-10-06

**Authors:** Jasmine Y. Serpen, Lev Prasov, Wadih M. Zein, Catherine A. Cukras, Denise Cunningham, Elizabeth C. Murphy, Amy Turriff, Brian P. Brooks, Laryssa A. Huryn

**Affiliations:** ^1^National Eye Institute, National Institutes of Health, Bethesda, MD 20892, USA; ^2^Case Western Reserve University School of Medicine, Cleveland, OH 44106, USA; ^3^Department of Ophthalmology and Visual Sciences, Kellogg Eye Center, University of Michigan, Ann Arbor, MI 48105, USA; ^4^Department of Human Genetics, University of Michigan, Ann Arbor, MI 48109, USA; ^5^The National Human Genome Research Institute, National Institutes of Health, Bethesda, MD 20892, USA

## Abstract

**Materials and Methods:**

Electronic medical records of patients evaluated in the Ophthalmic Genetics clinic at the National Eye Institute (NEI) between 2008 and 2018 were searched for a superficial ODD diagnosis. Color fundus and autofluorescence images were reviewed to confirm ODD, supplemented with optical coherence tomography (OCT) in uncertain cases when available. Demographic information, examination, and genetic testing were reviewed. Disc areas and disc-to-macula distance to disc diameter ratios (DM : DD) were calculated.

**Results:**

Fifty six of 6207 patients had photographically confirmed ODD (0.9%). Drusen were predominantly bilateral (66%), with a female (62%) and Caucasian (73%) predilection. ODD prevalence in our cohort of patients with inherited retinal degenerations was 2.5%, and ODD were more prevalent in the rod-cone dystrophy subgroup at 2.95% (OR = 3.3 [2.1–5.3], *P* < 0.001) compared to the ophthalmic genetics cohort. Usher patients were more likely to have ODD (10/132, 7.6%, OR = 9.0 [4.3–17.7], *P* < 0.001) and had significantly smaller discs compared to the rest of our ODD cohort (disc area: *P*=0.001, DM : DD: *P*=0.03). *Discussion*. While an association between ODD and retinitis pigmentosa has been reported, this study surveys a large cohort of patients with inherited eye conditions and finds the prevalence of superficial ODD is lower than that in the literature. Some subpopulations, such as rod-cone dystrophy and Usher syndrome, had a higher prevalence than the cohort as a whole.

## 1. Introduction

Optic disc drusen (ODD) are acellular deposits of calcified, proteinaceous material that are often found incidentally during ophthalmic examination and further confirmed with ancillary testing [1], including B-scan ultrasonography, fundus autofluorescence (FAF), and optical coherence tomography (OCT) [2]. Previous studies report an ODD prevalence ranging from 0.3 to 2.4% in the general population, occurring bilaterally in 75% of the cases [3]. The number and size of drusen are variable, and their location can be superficial or buried within the optic nerve head. ODD pathogenesis remains unclear but is likely multifactorial. Potential mechanisms include inherited optic disc dysplasia with blood supply compromise [4], abnormal optic disc vasculature, and alterations in axonal metabolism [5]. While patients with ODD are asymptomatic, enlargement or anterior migration of ODD can lead to thinning of the retinal nerve fiber layer and visual field defects. Additional complications include retinal vascular occlusions, choroidal neovascular membranes, and ischemic optic neuropathy [6].

Though a dominant mode of inheritance for ODD has been suggested [7, 8], most cases appear to be sporadic. There is a female and racial predilection [9], with Blacks having lower reported prevalence than other racial groups [10]. The considerable variability in prevalence estimates among different studies [11–13] may be related to ascertainment bias. Superficial ODD can be identified on exam or photographically, while buried ODD require supplemental imaging modalities for detection. Patients with ODD have been reported to have smaller and more crowded optic discs than controls [14]. ODD in otherwise clinically normal subjects have been associated with smaller optic disc area and shorter axial length [15]. In addition to disc area, the ratio of disc-to-macula distance to disc diameter (DM : DD) has been established as another independent assessment of disc size [16] but has not been evaluated in the context of ODD.

ODD have been found in the context of several ocular and systemic disorders, but it is unclear whether they are actually enriched in particular diagnoses [9]. An association between retinitis pigmentosa (RP) and ODD has been reported; a large cohort study found a prevalence of 10%, with no difference in frequency based on inheritance patterns [9, 17]. Among specific subtypes of RP, patients with Usher syndrome had an even higher prevalence of drusen; ODD were identified in 35% of type I and 8% of type II Usher syndrome patients [18]. RP and ODD can cooccur in an autosomal recessive syndrome caused by MFRP mutations, with other features including posterior microphthalmos and foveoschisis [7]. While associations between RP and ODD have been proposed, the burden of ODD across inherited ocular conditions is still unclear. In this study, we investigate the prevalence, demographic features, and optic disc parameters of ODD in a large cohort of patients with inherited eye conditions, including RP and Usher syndrome.

## 2. Materials and Methods

**Figure 1:**
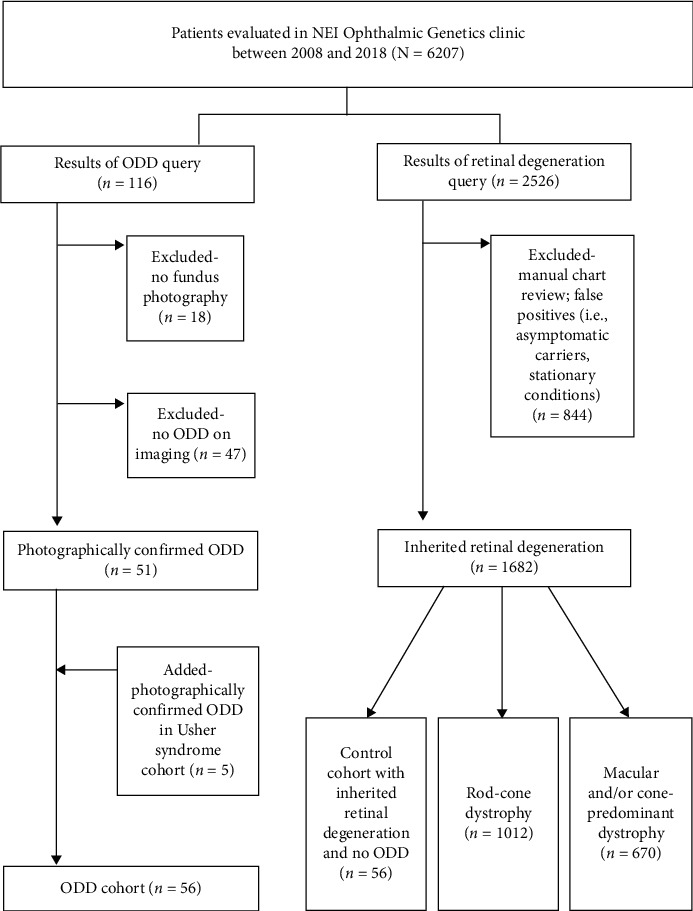
Flowchart depicting electronic medical record queries and validation.

A retrospective review of electronic medical records (EMR) was conducted for patients evaluated in the Ophthalmic Genetics and Visual Function Branch (OGVFB) clinic at the National Eye Institute (NEI) between 2008 and 2018 (Figure 1). All participants provided written, informed consent, and the study was approved by the Institutional Review Board of the NEI. All study protocols adhered to the tenets of the Declaration of Helsinki. Clinical assessments include a review of previous examination records, slit-lamp biomicroscopy, and a dilated funduscopic examination with or without retinal imaging based on examiners' judgment. Best-corrected visual acuity was measured using the Early Treatment of Diabetic Retinopathy Study [19] (ETDRS) chart recorded as Snellen Acuity or age-appropriate pediatric vision testing methods.

## 3. Medical Record Query and Verification

The following search terms were used to identify patients with ODD in the EMR: “optic disc drusen” OR “nerve head drusen” OR “ODD” OR “ONHD” OR “crowded optic disc” OR “optic nerve drusen” OR “disc drusen.” Charts were then manually reviewed for mention of ODD, and false positives were removed. For patients with available ophthalmic imaging, color fundus and fundus autofluorescence (FAF) images (Topcon, Tokyo, Japan; Spectralis; Heidelberg Engineering, Heidelberg, Germany; Optos, Dunfermline, Scotland) were reviewed by three independent graders to confirm the EMR ODD diagnosis, and in questionable cases, OCT (Cirrus HD-OCT, Carl Zeiss Meditec, Dublin, CA) of the optic nerve head was reviewed when available. Images were assessed for photographically apparent, superficial drusen; buried drusen were not included in this study. Color fundus images were reviewed alongside FAF to validate the presence of ODD; these were defined as hyper autofluorescent, reflective areas on the optic nerve on the FAF, corresponding to the photographically apparent ODD. Patients with poor quality imaging were excluded from subgroup analysis. An age- and gender-matched control population of 56 patients with a diagnosis of retinal degeneration without ODD and with sufficient quality retinal imaging was identified. Demographic information including sex, race, age, visual acuity, refractive error, laterality of drusen, diagnosis, and genetic testing results was retrieved from medical records (Figure 1).

To identify our inherited retinal degeneration (IRD) cohort among the ophthalmic genetics population, we used the following query search terms: “retinal degeneration” OR “cone-rod dystrophy” OR “cone-rod dystrophy” OR “rod-cone dystrophy” OR “rod-cone dystrophy” OR “retinal dystrophy” OR “RP” OR “RD” OR “XLRP” OR “pigmentary retinopathy” OR “retinitis pigmentosa” OR “macular degeneration” OR “cone dystrophy” OR “diminished ERG” OR “flat ERG” OR “unrecordable ERG” OR “Stargardt” OR “ABCA4” OR “salt-pepper retinopathy” OR “salt-pepper retinopathy.” Patient charts were manually reviewed for indication of a retinal degeneration as defined by a molecular or clinical phenotype of progressive inherited degeneration of the retina. Patients were then subdivided into the following subgroups: macular and/or cone-predominant dystrophy, rod-cone dystrophy, and Usher syndrome. The macular and/or cone-predominant dystrophy group included cone-rod dystrophy, cone dystrophy, maculopathy, Stargardt disease, pattern dystrophy, and vitelliform dystrophies as diagnoses. The rod-cone dystrophy group included RP, rod-cone dystrophy, pigmentary retinopathy, Leber congenital amaurosis, choroideremia, and gyrate atrophy but excluded Usher syndrome patients as they were a third subgroup used for analysis. Verification of IRD status was done via manual chart review for all patients identified from the EMR query. If retinal degeneration was not a definitive diagnosis but suspected, patients were included.

An additional query based on the following search terms was used to identify a subgroup of patients with Usher syndrome: “Usher syndrome” OR “Ush” OR “hearing loss” OR “hearing impairment” OR “deafness” OR “cochlear implant.” Charts were reviewed to confirm a molecular diagnosis and/or clinical phenotype of Usher syndrome. Patients with positive genetic testing results, defined as two pathogenic variants in Usher genes, were included. If genetic testing results were inconclusive or not available, judgment was made based on clinical data that demonstrated evidence of RP along with congenital or early-onset sensorineural hearing loss [20]. Cases of RP and hearing loss in which hearing loss was due to exposure or otherwise ambiguous were excluded. All available ophthalmic imaging was reviewed for this subset of patients to identify ODD.

## 4. Imaging Analysis

**Figure 2:**
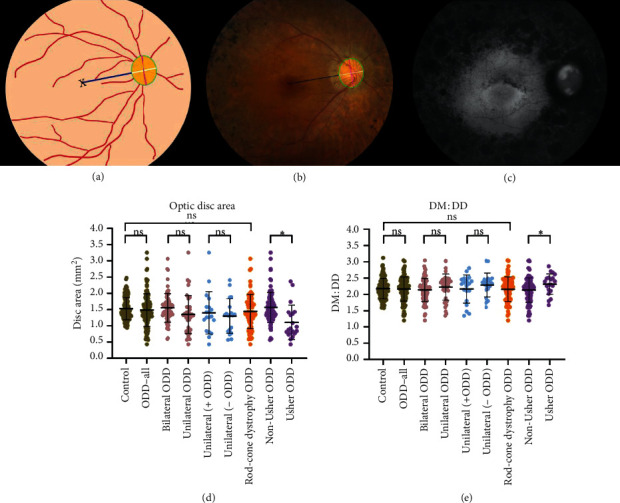
Optic disc parameters among the inherited retinal degeneration cohort. (a) Schematic diagram of optic disc parameters; black “x” indicates fovea centralis, taken as the macula, the navy line represents temporal edge of disc-to-macula distance, the white line indicates horizontal disc diameter, the magenta line indicates vertical disc diameter, and green circumference highlights optic disc area. Disc-to-macula distance (DM) was defined as the distance from the center of the disc to the macula, and disc diameter (DD) was defined as the average of the horizontal and vertical disc diameter. (b) Color fundus image of a patient with optic disc drusen (ODD) and retinitis pigmentosa, with measurements corresponding to schematic diagram in (a). (c) Fundus autofluorescence of the same patient with ODD and retinitis pigmentosa. Drusen are visualized as hyperautofluorescent areas on the optic disc. Box plots of (d) optic disc area and (e) DM : DD among each cohort and subgroup, with each individual data point representing one scored image. Patients with ODD and Usher syndrome had significantly smaller disc area and DM : DD than patients with ODD without Usher syndrome. ^*∗*^*P* < 0.05.

Optic disc size measurements were calculated in 110 eyes of 55 patients with ODD; one patient was excluded due to absent fundus imaging. Disc parameter measurements including disc area and DM : DD were made on standard, 50-degree field Topcon color fundus photos. Color fundus photo software (Merge Healthcare Solutions Inc., Hartland, WI) was used to measure vertical and horizontal disc diameters, DM distance, and disc area in both eyes of all patients. DM : DD ratios were calculated by dividing DM by the average of the horizontal and vertical disc diameters (Figures 2(a)–2(c)) [16]. DM distance was defined from the center of the optic disc to the fovea centralis.

## 5. Statistical Analysis

Differences between subgroups were evaluated with the two-tailed *t-*test or the Fisher exact test as indicated, and descriptive statistics were plotted using Graph Pad Prism (Graph Pad Software v 8.02, San Diego, California). Comparisons by the two-tailed *t-*test were made between the following groups: control vs. ODD, bilateral vs. unilateral ODD, pairwise between the eye with ODD and eye without ODD among the unilateral ODD group, rod-cone dystrophy ODD vs. control, and Usher syndrome ODD vs. non-Usher syndrome ODD. A significance level of *α* = 0.05 was used.

## 6. Results

Clinical records of 6207 patients with both developmental and degenerative phenotypes seen in the OGVFB clinic between 2008 and 2018 were systematically evaluated (Figure 1). Of the 1682 patients with a retinal degeneration, 1012 had a rod-cone dystrophy and 670 had a macular and/or cone-predominant dystrophy. Usher syndrome was present in 132 patients. In the overall ophthalmic genetics cohort (*N* = 6207), 116 individuals were identified as having possible ODD based on EMR search. Eighteen of these patients did not have adequate imaging for analysis, and 47 had no indication of ODD after review of charts and clinical imaging and were excluded. For the Usher syndrome cohort, all ophthalmic imaging was reviewed, and 5 additional patients with ODD were identified for a total of 56 patients with ODD in our ophthalmic genetics cohort (0.9%).

Examination of primary diagnosis for the 56 patients with ODD indicated retinal degeneration as the most common diagnosis (75%). Based on genetic testing results and clinical phenotype, syndromic ocular conditions accounted for 38% of this subgroup, isolated ocular conditions 50%, and the remaining 12% represent genetic conditions for which ophthalmic evaluations were requested. Thirty-six patients had a rod-cone dystrophy, 6 had a macular and/or cone-predominant dystrophy, and 14 patients had a genetic condition without a retinal degeneration (e.g., xeroderma pigmentosum, DICER1 syndrome, and Ehlers–Danlos syndrome) (Table 1).

Other demographic features of our ODD cohort were consistent with previously published reports [3, 9, 13]. ODD were predominantly bilateral (66%), a female predilection was noted (62%), and 73% was Caucasian (Table 2).

Demographic features in our control group closely matched those of the ODD cohort in age, sex, race, and refractive error (Table 2). The prevalence of ODD in the 1682 patients with an IRD was 2.5%, but ODD were more prevalent in the rod-cone dystrophy subgroup at 2.95% (26 out of 880 patients, OR = 3.3, 95% CI = 2.1–5.3, *P* < 0.001) compared to the general ophthalmic genetics cohort. Likewise, a higher proportion of ODD patients had a rod-cone dystrophy versus macular and/or cone-predominant dystrophy as per Fisher's exact test (*P*=0.006). The prevalence of Usher syndrome within the ODD cohort was 18% (*n* = 10/56), and 10/132 Usher syndrome patients had ODD (OR = 9.0 compared to the general ophthalmic genetics cohort, 95% CI = 4.3–17.7, *P* < 0.001).

A rhodopsin (*RHO*) mutation was found in 5 of 26 (19%) rod-cone dystrophy ODD patients. The Usher syndrome patients had mutations in several Usher genes including *ADGRV1*, *CLRN1*, *USH2A*, *CDH23*, and *MYO7A.* Mutations in *CDH23* and *MYO7A* were the most common in our cohort, accounting for 50% of the Usher syndrome ODD patients (Table 3).

Disc parameters have been variably associated with prevalence of ODD in the general population [21]. Small scleral canals may be associated with ODD, causing physical compression, blocking axoplasmic flow, and leading to axonal degeneration and retinal ganglion cell damage [22]. Evaluation of scleral canal size based on fundus photography has shown an association between a small scleral canal and vascular anomalies in ODD patients [21], while other studies based on OCT have had mixed results [22, 23]. We explored this in our retinal degeneration cohort by measuring optic disc area and DM : DD in patients with ODD and in a demographically matched control cohort with retinal degeneration alone. There were no statistically significant differences in optic disc area or DM : DD between retinal degeneration patients with and without ODD or in subgroup analysis with rod-cone dystrophy patients (Figures 2(d)–2(e)). However, ODD patients with Usher syndrome had significantly smaller discs based on area and DM : DD (mean 1.10 ± 0.5 mm^2^ and 2.32 ± 0.3) compared to the remaining ODD cohort (disc area: *P*=0.001, DM : DD: *P*=0.03, *t*-test) (Table S1).

## 7. Discussion

We present the largest cohort of patients with inherited eye conditions and ODD. We found similar demographic patterns of ODD as compared to the literature, with a female and Caucasian predilection [9, 13]. There has been a reported association of ODD and RP although there is broad variation in prevalence (1.4–80.0%) for clinically reported cases [17]. This study represents one of the most comprehensive evaluations of detailed clinical information and optic disc parameters in patients with ODD and inherited eye conditions and is novel in its assessment of genetic diagnoses and independent indicators of optic disc size.

Our findings indicate that the ODD prevalence in patients with inherited eye conditions (0.9%) is on the lower end of the range posited in the literature for the general population (0.3–2.4%) though these estimates often include buried drusen [3]. The highest estimates of ODD (2–2.4%) are based on retrospective analysis of necropsy [24], while noninvasive imaging with color fundus photography, B-scan, OCT, and autofluorescence yielded lower estimates [13, 25]. It is likely that many of our imaging modalities underestimate the true ODD prevalence, especially as fundus photography is limited in detection of buried drusen [25]. The reported increased prevalence of ODD in RP could also be secondary to small cohorts of RP patients or single case reports [7, 26–29]. Grover et al. reported an ODD prevalence of 9.2% in patients with RP, identified by color fundus photography [17]. Likewise, we observed a higher prevalence of ODD in our RP/rod-cone dystrophy cohort relative to the cohort as a whole (3.6% versus 0.9%).

The discrepancies in the prevalence between our findings and those of previous reports could be explained in two ways. First, different cohorts of RP patients may have different rates of ODD. Grover et al. [17] excluded patients with syndromes associated with an RP-like retinal disease, such as Bardet–Biedl syndrome, which were included in our rod-cone dystrophy cohort. Furthermore, while we defined patients in our cohort by genetic diagnosis, this information was not available in prior studies. Second, previous large studies relied on fundus imaging for identification of drusen, which may have led to an overestimation of drusen in anomalous appearing optic discs. In our study, adjunctive use of FAF and OCT improved our ability to distinguish ODD from glial tufts and optic disc anomalies. The use of additional imaging modalities may augment our ability to accurately identify photographically apparent ODD, therefore improving our prevalence estimates. Overall estimates may be still underrepresented in our study, as we did not include buried drusen. Given our reliance on EMR data and presence of imaging, there is the potential for clinical underreporting of ODD in the setting of complex inherited ocular conditions. Nonetheless, our finding that ODD are enriched in the rod-cone dystrophy and Usher syndrome cohorts is supportive of prior reports [17, 18]. We demonstrate that macular and/or cone-predominant disorders do not have an increased association with ODD as compared to the general population.

On fundus photography, patients with ODD have smaller optic disc size than controls as indicated by disc area and horizontal diameter [14]. While smaller discs have been associated with ODD in the general population [21, 30], the disc size as measured by area and DM : DD was similar in patients with ODD as compared to our representative retinal degeneration control subgroup. These results suggest that disc size is not associated with a predisposition to ODD in our cohort. The proposition that ODD is more common in small discs [31] may be confounded by an alternative relationship—the drusen themselves may lead to mechanical strain including axonal fiber crowding and compression of surrounding vasculature [32], which could be contributing to optic disc congestion. However, it remains possible that the mechanism for drusen formation in the setting of inherited eye disease may differ from the general population, for instance, diminished ganglion cell functionality, rather than a small, crowded disc.

Approximately 30% of families with autosomal dominant RP have mutations in the *RHO* gene [33], and a few mutations have been implicated in autosomal recessive RP [34]. Rhodopsin is expressed only in rod photoreceptors [33] and not the optic disc. This suggests that the mechanism for ODD formation in RP may be related to the retinal degeneration process itself, rather than optic disc size or specific proteins in the optic nerve head. On the other hand, ODD are much more prevalent in Usher syndrome patients, and these patients also had smaller optic discs. Usher syndrome is a ciliopathy or disorder caused by a defect in ciliary protein trafficking [35], and many of the Usher gene protein products, including myosin VIIa, cadherin 23, usherin, and harmonin, are thought to be expressed in retinal ganglion cells [36]. Disruption of these proteins involved in synapse function and transport may share a mechanistic basis with the disruption of axoplasmic flow responsible for ODD formation, though further investigation is needed. Other ciliopathies in our rod-cone dystrophy cohort including Bardet–Biedl syndrome, Leber congenital amaurosis, and Joubert syndrome do not have an overrepresentation of ODD, suggesting that Usher gene products may have unique roles in the optic nerve head and pathogenesis of drusen.

A potential association between ODD and glaucoma has previously been explored, as both female-predominant conditions [37] have a hereditary component and are associated with optic disc changes. In a prospective evaluation using a patient-directed survey, incidence of glaucoma in patients with ODD was 20.7% compared to 2.8% in healthy controls [38]. The frequency of visual field loss is higher in eyes with ODD and ocular hypertension [39]. Distinguishing glaucoma from ODD as the underlying etiology of visual field defects presents a formidable challenge. In our cohort, we did not have a large proportion of patients with clinically apparent glaucoma, but we were limited in visual field evaluation given the high proportion of retinal degeneration in our patients. Nonetheless, commonalities in the pathogenesis of ODD and glaucoma present an interesting realm for future investigation in other cohorts.

Limitations of our study include exclusion of buried drusen. The functional consequences of ODD in our cohort were difficult to ascertain given the confounding factor of visual acuity and visual field impairment due to the underlying retinal degeneration. While our cohort represents the largest to date, its derivation from the Ophthalmic Genetics clinic at the NEI limits the generalizability of our results. This study did not include a general population comparison group, and as such the findings are interpretable only in the context of the evaluated retinal degenerations.

Our work suggests that the rate of superficial ODD in inherited ocular conditions is lower than previously reported though some subpopulations such as rod-cone dystrophy and Usher syndrome have a higher prevalence as compared to a general ophthalmic genetics cohort. As such, anomalous or elevated discs warrant careful clinical evaluation and should not be attributed to ODD alone. Visual field changes observed in these patients may not be consistent with retinal degeneration progression, and workup is recommended to rule out other high-risk causes of disc elevation such as increased intracranial pressure or infiltrative processes. Future studies may help elucidate the mechanism of drusen formation and pathogenesis. Understanding the role of proteins implicated in Usher syndrome may help establish a causal link between ODD and Usher syndrome. This future work may also uncover the broader mechanisms of drusen formation and pathogenesis in the general population.

## Figures and Tables

**Table 1:** ODD cohort diagnoses.

Diagnosis	Number of ODD patients (%)
Retinal degeneration	42 (75)
Rod-cone dystrophy	36 (64)
Macular and/or cone-predominant dystrophy	6 (11)
Genetic condition without a retinal degeneration	14 (25)

Isolated ocular condition	28 (50)
Syndromic ocular condition	21 (38)
Genetic conditions for which ophthalmic evaluations were requested	7 (12)

**Table 2:** Demographic and ophthalmic characteristics of ODD and control patients.

	ODD—all (*n* = 56)	ODD—unilateral (*n* = 19)	ODD—bilateral (*n* = 37)	Control (*n* = 56)
Sex				
Male	21 (38%)	7 (37%)	14 (38%)	30 (54%)
Female	35 (62%)	12 (63%)	23 (62%)	26 (46%)

Age (mean ± SD, years)	32.9 ± 17.5	30.7 ± 18.5	34.1 ± 17.1	38.6 ± 17.8

Age (range, years)	5.0–73.0	5.0–69.0	8.0–73.0	3.0–84.0

Race				
Caucasian	41 (73%)	14 (74%)	27 (73%)	28 (50%)
AA	2 (4%)	1 (5%)	1 (3%)	15 (27%)
Others	13 (23%)	4 (21%)	9 (24%)	13 (23%)

LogMAR BCVA (range)				
OD	−0.097-LP	0.000–1.204	−0.097-LP	−0.097-LP
OS	−0.097-NLP	0.000-CF	−0.097-NLP	−0.204-LP

Refractive error (median, IQR, D)				
OD	−1.00 (2.88)	−1.63 (2.16)	−0.38 (2.38)	−1.63 (3.88)
OS	−0.63 (3.50)	−1.88 (4.25)	−0.25 (3.00)	−1.38 (3.53)

SD: standard deviation; AA: African American; logMAR BCVA: logarithm of the minimal angle of resolution best-corrected visual acuity; OD: right eye; OS: left eye; LP: light perception; NLP: no light perception; CF: count fingers; IQR: interquartile range; D: diopter.

**Table 3:** Usher cohort genetic testing results.

Usher gene	Number of patients (*n* = 132) (%)	Number of ODD patients (*n* = 56) (%)
*USH2A*	55 (42)	2 (4)
*MYO7A*	17 (13)	2 (4)
*CDH23*	10 (8)	3 (5)
*CLRN1*	8 (6)	1 (2)
*USH1C*	7 (5)	0
*ADGRV1*	6 (5)	1 (2)
*PCDH15*	5 (4)	0
*USH1G*	2 (2)	0
Multiple genes	6 (5)	0
Clinical phenotype	16 (12)	1 (2)

## Data Availability

The data used to support the findings of this study are available from the corresponding author upon request.

## Supplementary Materials

Supplementary Materials Table S1: disc area and DM : DD measurements among the ophthalmic genetics population.
