# Supplementary material for: Clinical Features of Optic Disc Drusen in an Ophthalmic Genetics Cohort
Source: J Ophthalmol. 2020 Oct 6;2020:5082706. doi: 10.1155/2020/5082706 (PMC7557906; doi:10.1155/2020/5082706)
Supplement: Supplementary Materials — Table S1: disc area and DM : DD measurements among the ophthalmic genetics population. [file 5082706.f1.docx]

**Supplemental Table 1. Disc Area and DM:DD Measurements Among the Ophthalmic Genetics Population**

|  | **Disc Area (mean ± SD, mm^2^)** | **DM:DD (mean ± SD)** |
| --- | --- | --- |
| **Control (n=56)** | 1.5 ± 0.3 | 2.7 ± 0.3 |
| **ODD (n=55*)** | 1.5 ± 0.5 | 2.6 ± 0.4 |
| **Bilateral (n=36)** | 1.6 ± 0.4 | 2.6 ± 0.3 |
| **Unilateral (n=19)** | 1.4 ± 0.6 | 2.7 ± 0.4 |
| **+ ODD** | 1.4 ± 0.7 | 2.6 ± 0.4 |
| **- ODD** | 1.3 ± 0.5 | 2.8 ± 0.4 |
| **RCD ODD (n=36)** | 1.4 ± 0.5 | 2.6 ± 0.4 |
| **Non-Usher ODD (n=45)** | 1.6 ± 0.5 | 2.6 ± 0.4 |
| **Usher ODD (n=10)** | 1.1 ± 0.5 | 2.8 ± 0.3 |
|  |  |  |

*1 patient excluded due to lack of color fundus imaging, DM:DD: disc-to-macula distance divided by average of vertical and horizontal disc diameters, RCD: rod-cone dystrophy
